# Primary Peritoneal High-Grade Serous Carcinoma Presenting With Diffuse Carcinomatosis and No Ovarian Mass: A Case of Exceptional Response

**DOI:** 10.7759/cureus.105776

**Published:** 2026-03-24

**Authors:** Eliany Leon Figueredo, Miguel Angel Del Sol Pedraza, Lismabel Marin Gonzalez, Eduardo Oropesa, Vivian Varona Rodriguez, Osmany Reyes Luaces

**Affiliations:** 1 Internal Medicine, Englewood Health Physician Network, New Jersey, USA; 2 Vascular Surgery, Memorial Hermann Health System, Texas, USA; 3 Family Medicine, Ideal Care Clinic, Texas, USA; 4 Dermatology, Advanced Dermatology and Cosmetic Surgery, Lehigh Acres, USA; 5 Family Medicine, Riverside Family and Lifestyle Medicine, Cape Coral, USA; 6 Nephrology, Hospital Hermanos Ameijeiras, Havana, CUB

**Keywords:** ascitic fluid cytology, high-grade serous carcinoma, neoadjuvant chemotherapy, pathologic complete response, peritoneal carcinomatosis, primary peritoneal carcinoma

## Abstract

Primary peritoneal serous carcinoma (PPSC) is a rare Müllerian malignancy that closely resembles ovarian carcinoma but is distinguished by minimal or absent ovarian involvement despite extensive peritoneal disease. Diagnosis becomes particularly challenging when no adnexal mass is identified, and in such cases, ascitic fluid cytology with Müllerian immunophenotyping may provide the only definitive diagnostic pathway. We report the case of a 63-year-old woman who presented with worsening abdominal pain, diffuse ascites, and imaging findings consistent with peritoneal carcinomatosis but without an ovarian or pelvic mass. Ascitic fluid cytology repeatedly demonstrated malignant epithelial cells expressing PAX8, CK7, and abnormal p53, confirming Müllerian origin and supporting a diagnosis of PPSC. Serial CA-125 levels rose markedly prior to treatment initiation, reaching 864.8 U/mL. The patient received neoadjuvant chemotherapy with carboplatin, paclitaxel, and bevacizumab, resulting in rapid symptomatic improvement, normalization of CA-125, and complete radiologic resolution of carcinomatosis. She subsequently underwent interval debulking surgery, during which no residual viable carcinoma was identified, consistent with a pathologic complete response. This case illustrates the diagnostic challenges of PPSC in the absence of an adnexal mass and demonstrates the value of ascitic cytology and Müllerian immunoprofiles when tissue biopsy is not feasible. It also highlights the prognostic significance of robust chemotherapy response, as pathologic complete remission remains uncommon in high-grade serous Müllerian carcinomas. Overall, this case underscores the importance of recognizing atypical presentations of PPSC and shows that exceptional therapeutic outcomes are achievable with appropriately selected neoadjuvant therapy.

## Introduction

Primary peritoneal serous carcinoma (PPSC) is a rare epithelial malignancy arising from the peritoneal lining that was first described by Swerdlow in 1959 as a “mesothelioma of the pelvic peritoneum.” Since then, it has been referred to by several names, including extraovarian primary peritoneal carcinoma, psammoma carcinoma, and primary peritoneal carcinoma. It has an estimated incidence of 6.78 cases per million people, primarily affecting women over the age of 60, and is rarely observed in men [1].

PPSC shares many clinical, histologic, and molecular features with high-grade serous ovarian carcinoma, which makes the diagnosis clinically challenging [2]. However, according to the diagnostic criteria established by the Gynecologic Oncology Group (GOG), PPSC is distinguished by minimal or absent ovarian involvement despite extensive peritoneal disease. Specifically, the ovaries must be either normal in size or demonstrate only superficial tumor involvement measuring ≤5 mm, while the bulk of disease is located within the peritoneum, allowing differentiation from advanced ovarian carcinoma with secondary peritoneal dissemination. Histologically, PPSC is generally classified into two serous subtypes: high-grade serous carcinoma (HGSC) and low-grade serous carcinoma (LGSC), with HGSC accounting for approximately 85-90% of serous carcinomas [2,3].

When a primary mass cannot be identified on imaging or at surgery, a diagnosis can be established through cytologic evaluation of ascitic fluid supported by immunohistochemistry [4]. Malignant cells in ascitic fluid that express Müllerian markers, such as PAX8 and CK7, along with abnormal p53, strongly support HGSC of peritoneal origin and help exclude non-Müllerian malignancies. Although establishing this diagnosis solely by cytology and immunoprofile without a detectable mass is uncommon, it enables earlier recognition and management in atypical presentations [5].

The prognosis of HGSC is generally unfavorable, as most patients present with advanced-stage disease at diagnosis [2]. However, response to platinum-based neoadjuvant chemotherapy is a key prognostic factor: significant CA-125 decline and strong histopathologic response correlate with improved progression-free survival (PFS) and overall survival (OS), reflecting tumor chemosensitivity [6].

We present a case of diffuse peritoneal carcinomatosis without an ovarian mass, meeting GOG diagnostic criteria for PPSC, in which diagnosis was established through repeated positive ascitic cytology and Müllerian immunophenotyping. The case is notable for an exceptional response to neoadjuvant chemotherapy, with marked CA-125 normalization that allowed successful surgical intervention. It underscores several important diagnostic challenges, including the absence of a detectable primary mass, dependence on cytologic evaluation for diagnosis, and the need to distinguish PPSC from advanced ovarian carcinoma, highlighting its clinical and educational value.

## Case presentation

A 63-year-old woman with a past medical history of hypertension, hyperlipidemia, and a benign left breast tumor excised in 2016 presented to the emergency department with acute worsening of intermittent sharp lower abdominal pain over one week. She reported experiencing intermittent abdominal discomfort over the preceding several months, unrelieved by laxatives or famotidine. She was hemodynamically stable on arrival. A bladder scan suggested >800 mL of urinary retention, although Foley catheterization drained only 100 mL, after which her pain improved. The discrepancy was attributed to tense ascites rather than true urinary retention.

Four months prior to presentation, during evaluation of intermittent abdominal symptoms, a pelvic transabdominal and transvaginal ultrasound demonstrated a heterogeneous, myomatous uterus with multiple fibroids, but no adnexal mass or free pelvic fluid. The endometrium was obscured, and both ovaries were nonvisualized. No cystic structures were identified at that time.

Initial laboratory evaluation demonstrated hypoalbuminemia, mild anemia, thrombocytosis, and a markedly elevated CA-125, with otherwise unremarkable chemistry and tumor markers. Urinalysis showed mild pyuria and proteinuria, and urine culture grew *Staphylococcus epidermidis* at a low colony count, considered likely contamination. Abnormal diagnostic findings are summarized in Table 1.

**Table 1:** Initial abnormal laboratory and urinalysis findings at presentation CA-125: Cancer antigen 125, CFU/mL: colony-forming units per milliliter, g/dL: grams per deciliter, mg/dL: milligrams per deciliter, %: percent, ×10⁹/L: billions per liter, U/mL: units per milliliter

Test	Result	Reference Range	Interpretation
Albumin	3.0 g/dL	3.5–5.0 g/dL	Low
Total Protein	5.3 g/dL	6.0–8.3 g/dL	Low-normal
Glucose	101 mg/dL	70–99 mg/dL	Mildly elevated
Hemoglobin	11.0 g/dL	12–16 g/dL	Mild anemia
Hematocrit	32.7%	36–46%	Low
Platelets	518 ×10⁹/L	150–400 ×10⁹/L	Thrombocytosis
CA-125	451.7 U/mL	<35 U/mL	Markedly elevated
Urine Color	Dark yellow	Yellow	Abnormal
Urine Clarity	Cloudy	Clear	Abnormal
Urine Protein	100 mg/dL	Negative/Trace	Proteinuria
Urine Blood	Trace	Negative	Abnormal
Leukocyte esterase	Small	Negative	Pyuria
Urine Bilirubin	Small	Negative	Abnormal
Urine Ketones	15 mg/dL	Negative	Abnormal
Urine Culture	80,000 CFU/mL Staph. epidermidis	—	Likely contaminant

**Figure 1:**
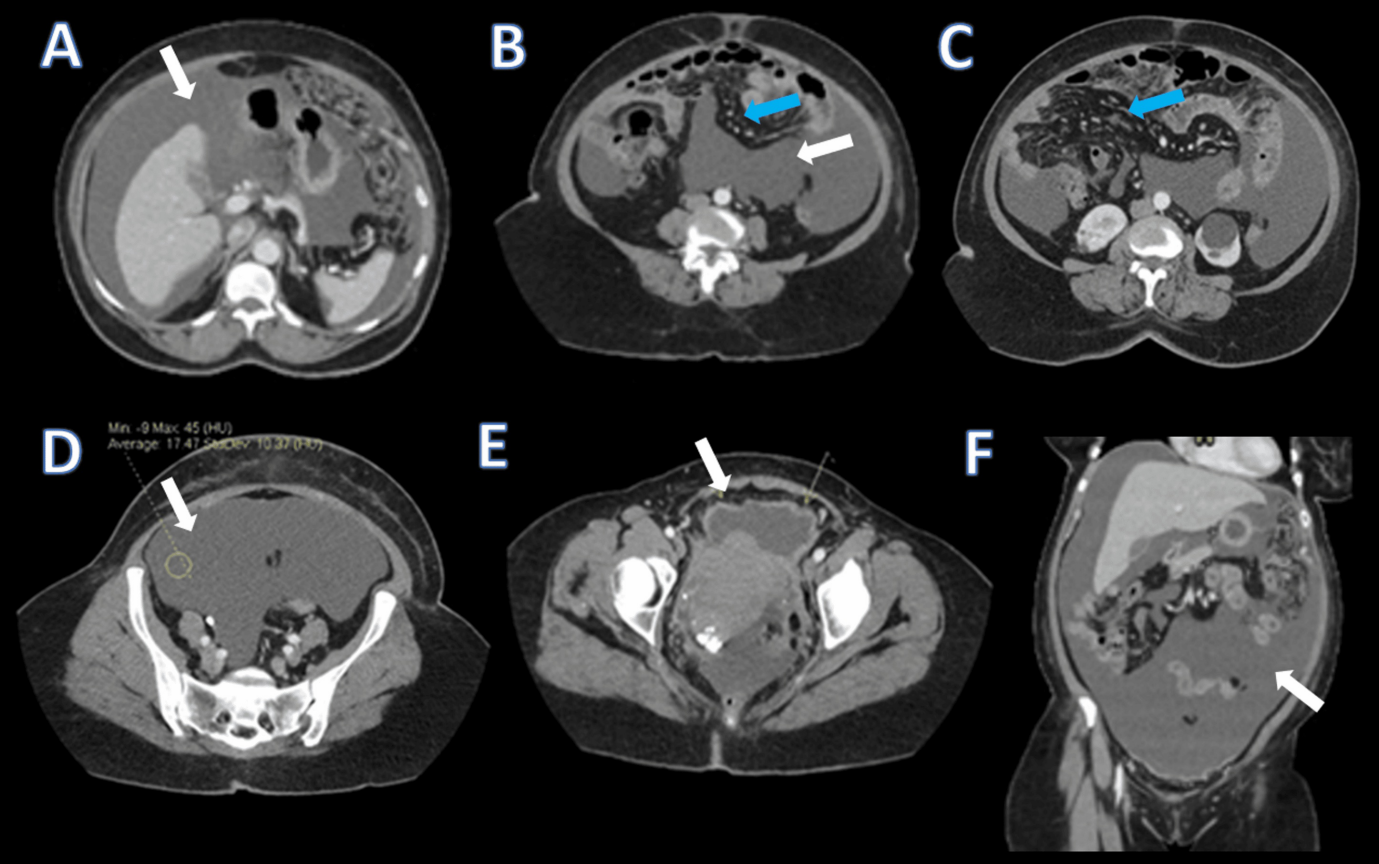
Contrast-enhanced CT of the abdomen and pelvis demonstrating diffuse peritoneal disease (A–C) Axial contrast-enhanced CT images of the upper and mid abdomen showing moderate-to-large volume ascites (white arrows) with diffuse omental and mesenteric reticulation consistent with omental caking (blue arrows). (D–E) Axial pelvic CT images demonstrating large-volume ascites and peritoneal thickening along the anterior pelvic peritoneum (white arrows), without evidence of an adnexal mass. (F) Coronal reconstruction demonstrating extensive intraperitoneal fluid distribution (white arrow) and diffuse peritoneal involvement.

Computed tomography (CT) abdomen/pelvis with intravenous (IV) contrast was obtained as the initial imaging modality, given its rapid availability and established role as the first-line study in the acute evaluation of intra-abdominal pathology. Imaging demonstrated interval development of moderate-to-large ascites with peritoneal thickening and enhancement, most prominent along the anterior inferior peritoneum. There was diffuse, thick omental and mesenteric reticulation consistent with metastatic caking, as well as gastric mural prominence suggestive of possible serosal implants. No adnexal mass or focal solid-organ lesions were identified. Additional findings included multiple calcified and non-calcified uterine fibroids, small bilateral pleural effusions, and no enlarged lymph nodes (Figure 1).

**Figure 2:**
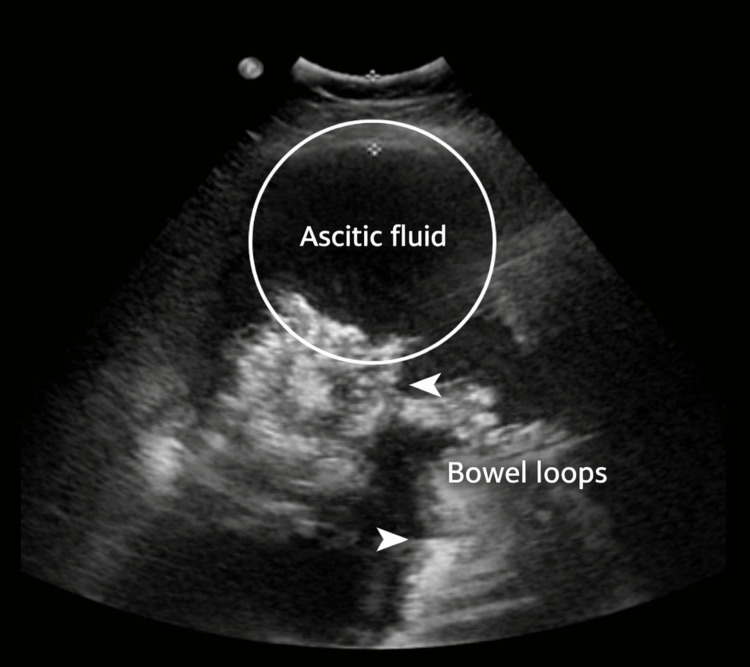
Ultrasound-guided paracentesis Transabdominal ultrasound of the left lower quadrant demonstrating a large anechoic pocket of intraperitoneal fluid consistent with ascites (circled). The surrounding echogenic structures correspond to displaced bowel loops within the peritoneal cavity. The fluid-filled space provided an appropriate window for ultrasound-guided paracentesis.

Ultrasound-guided paracentesis removed 3 liters of yellow ascitic fluid (Figure 2). Fluid analysis showed serum-ascites albumin gradient (SAAG) <1.1 g/dL and protein >4.5 g/dL, consistent with exudative ascites. Cytology was positive for malignant adenocarcinoma cells, and immunohistochemistry demonstrated positivity for PAX8, CK7, and p53, and negativity for ER, CK20, and CDX2, supporting a diagnosis of PPSC.

In outpatient follow-up with gynecologic oncology, the patient was assessed as likely having stage III primary peritoneal carcinoma, given the absence of an ovarian or pelvic mass despite diffuse peritoneal involvement. Repeat ascitic cytology continued to show malignant adenocarcinoma with a Müllerian immunoprofile (PAX8+, CK7+, p53+, ER-, CK20-, CDX2-), supporting the diagnosis of PPSC. Given the absence of a discrete, safely accessible solid lesion and the consistent cytologic and immunohistochemical findings across repeated samples, a peritoneal biopsy was not performed, and the diagnosis was established based on ascitic cytology and Müllerian immunophenotyping.

**Figure 3:**
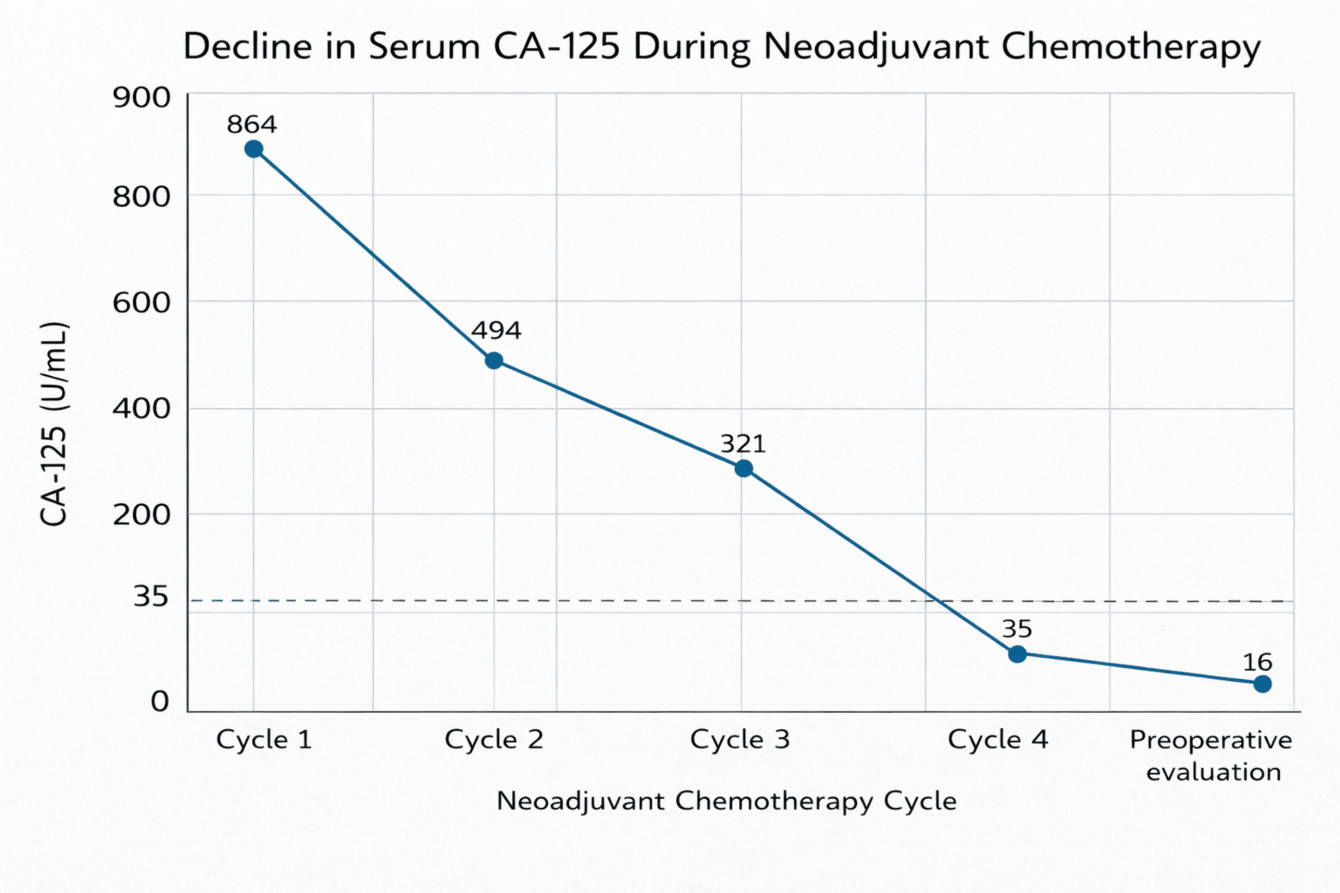
Decline in serum CA-125 levels during neoadjuvant chemotherapy Serial measurements of serum CA-125 demonstrate a marked biochemical response to neoadjuvant chemotherapy. Levels decreased progressively from 864 U/mL at treatment initiation to within the normal range after four cycles (16 U/mL) prior to interval cytoreductive surgery. The dashed horizontal line indicates the upper limit of the normal reference range. CA-125: Cancer antigen 125 U/mL: Units per milliliter

Prior to treatment initiation, serial CA-125 values demonstrated a progressive rise over approximately 4-6 weeks, from 451.7 U/mL at admission to 864.8 U/mL at the start of neoadjuvant therapy. She subsequently began neoadjuvant chemotherapy with a carboplatin-paclitaxel regimen, with bevacizumab included during the early cycles. Over the course of approximately three months, she completed four cycles of systemic therapy prior to planned interval cytoreductive surgery. The treatment was well-tolerated, without significant adverse effects. During therapy, the patient experienced marked symptomatic improvement, including resolution of abdominal pain and progressive reduction of ascites, which correlated with treatment response. Following the initiation of therapy, CA-125 demonstrated an excellent biochemical response, declining from 864.8 U/mL to within the normal range after four cycles (Figure 3). By the time of surgical planning, the patient was asymptomatic, and interval imaging was arranged to confirm treatment response prior to proceeding with cytoreductive surgery.

**Figure 4:**
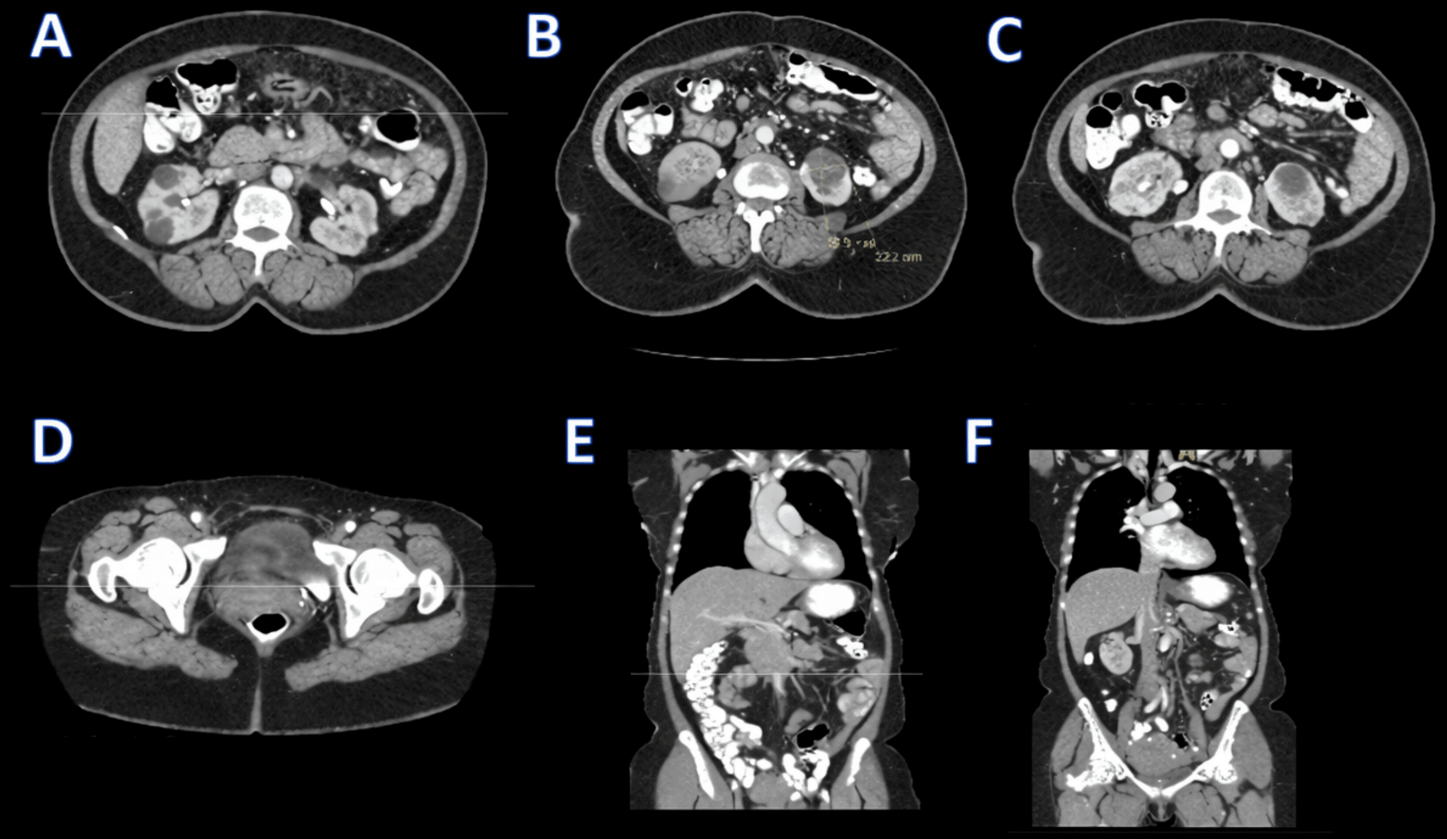
Post-treatment contrast-enhanced CT demonstrating complete radiologic resolution of prior peritoneal disease (A–C) Axial contrast-enhanced CT images of the abdomen demonstrating normal appearance of the liver, bowel, mesentery, and omentum without evidence of ascites, peritoneal thickening, or omental caking. (D) Axial CT image of the pelvis demonstrating normal uterus and adnexa without pelvic masses or free fluid. (E–F) Coronal CT reconstructions of the abdomen and pelvis confirming the absence of ascites, lymphadenopathy, peritoneal disease, or solid-organ masses.

Preoperative CT of the chest, abdomen, and pelvis showed no thoracic or abdominopelvic lymphadenopathy, no effusions, no ascites, and no masses in the lungs, solid organs, uterus, or adnexa. The bowel and peritoneum appeared normal, with complete resolution of previously seen omental and peritoneal disease. No residual neoplasm was identified. Compared with the prior CT, which demonstrated ascites and diffuse omental carcinomatosis, this scan showed complete interval resolution of all malignant findings (Figure 4).

She then underwent robotic total hysterectomy, bilateral salpingo-oophorectomy, omentectomy, and tumor debulking. Intraoperatively, the uterus was enlarged to approximately the 14-week size due to fibroids. Multiple subcentimeter peritoneal implants were identified along the small- and large-bowel mesentery and pelvic peritoneum, and the omentum was thickened and adherent to the mesentery, consistent with treatment effect. Both ovaries and fallopian tubes appeared grossly normal with no adnexal mass. Estimated blood loss was 20 mL, and peritoneal washings were obtained for cytology.

**Figure 5:**
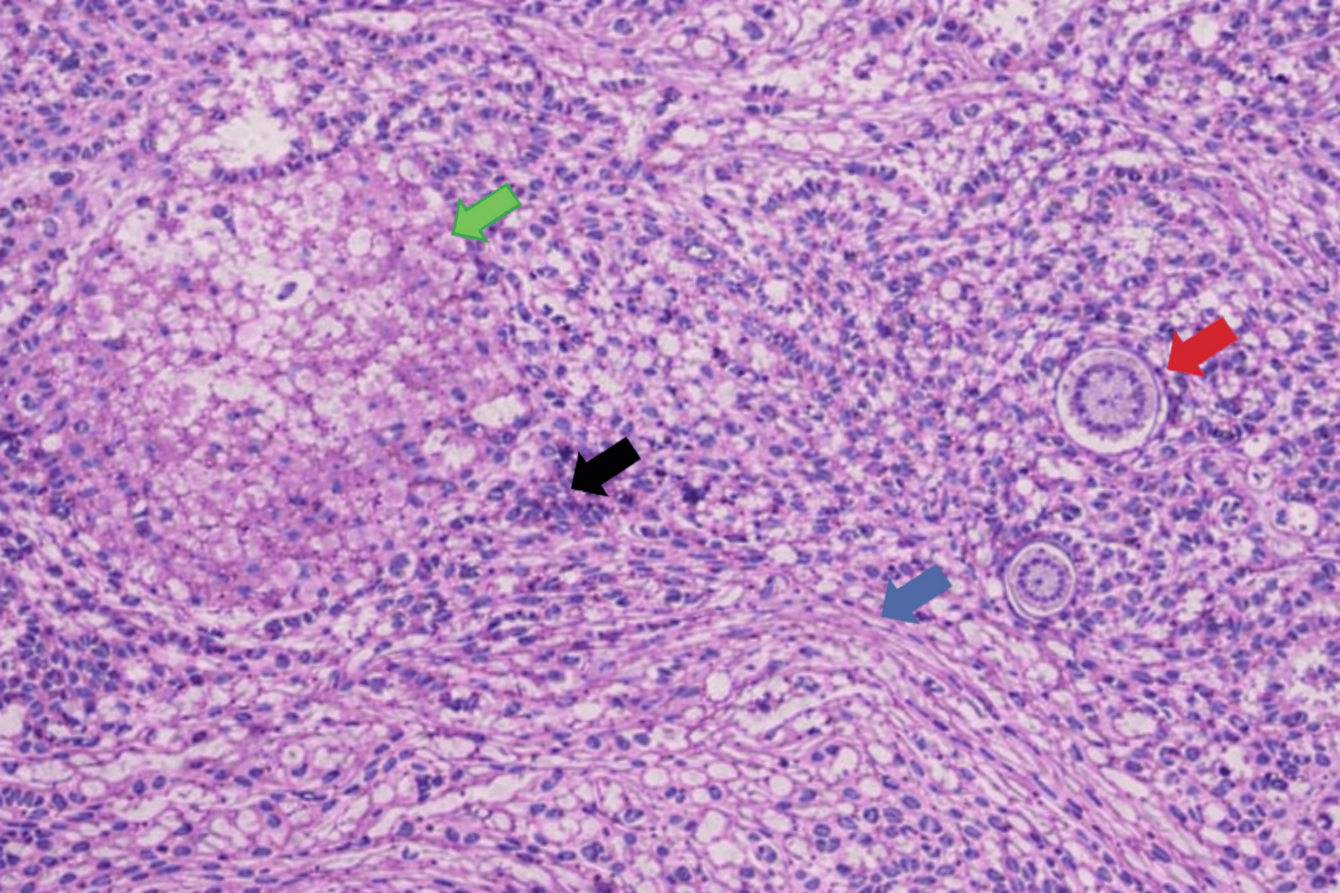
Histopathologic findings demonstrating treatment effect and pathologic complete response after neoadjuvant chemotherapy Representative H&E section showing treatment-related changes characterized by tumor necrosis (green arrow), lymphocytic inflammatory infiltrate (black arrow), stromal fibrosis (blue arrow), and psammomatous calcifications (red arrow). No residual viable carcinoma is identified. These findings are consistent with a pathologic complete response to neoadjuvant therapy. H&E stain, original magnification ×200. H&E: Hematoxylin and eosin

**Figure 6:**
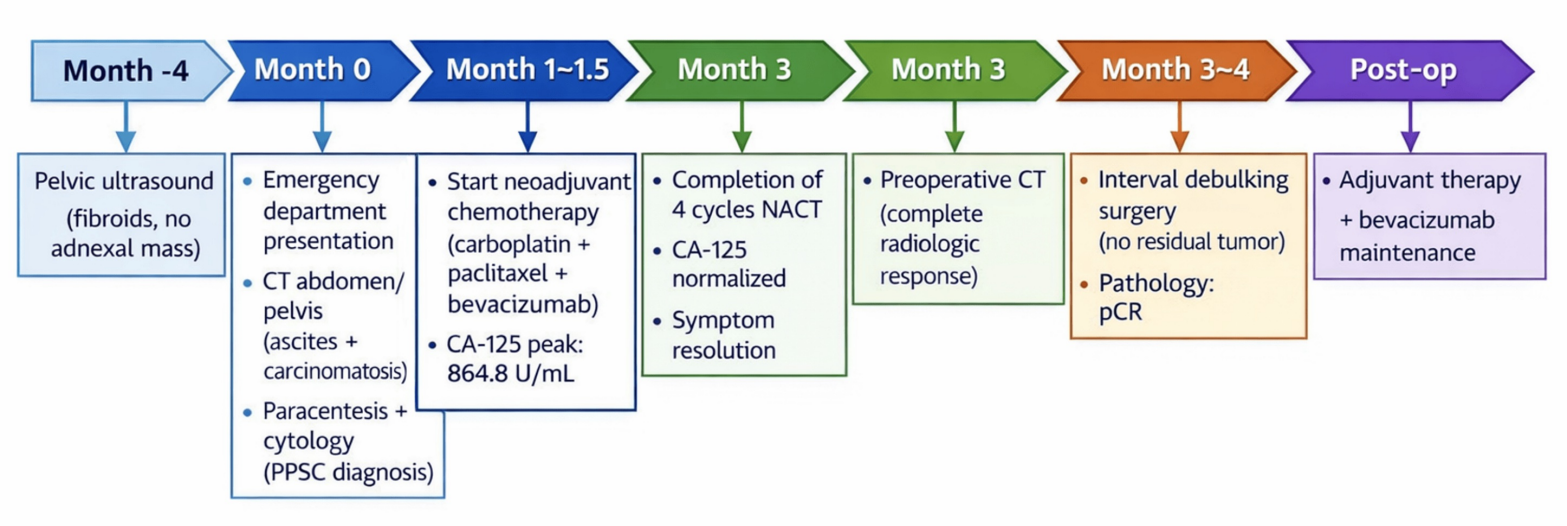
Timeline of clinical presentation, diagnostic evaluation, and therapeutic management Timeline illustrating the patient’s clinical course from initial presentation to post-operative management. Key milestones include prior pelvic ultrasound, emergency department presentation, diagnostic imaging, cytologic confirmation of Müllerian carcinoma, initiation and completion of neoadjuvant chemotherapy, normalization of CA-125 levels, radiologic resolution of disease, and interval cytoreductive surgery demonstrating pathologic complete response, followed by adjuvant therapy and maintenance treatment. PPSC: primary peritoneal serous carcinoma; CT: computed tomography; NACT: neoadjuvant chemotherapy; CA-125: cancer antigen 125; pCR: pathologic complete response

Final pathology demonstrated no residual viable carcinoma in the uterus, adnexa, omentum, or peritoneum. All specimens showed only fibrosis, necrosis, lymphocytic infiltrate, and scattered psammomatous calcifications, consistent with a pathologic complete response to neoadjuvant therapy (Figure 5). Peritoneal washings were negative for malignant cells. She recovered well without complications and subsequently proceeded with the planned adjuvant regimen, including continuation of carboplatin and paclitaxel with transition to bevacizumab maintenance therapy, followed by routine surveillance. A comprehensive timeline summarizing the patient’s clinical course, including key diagnostic, therapeutic, and response milestones, is provided in Figure 6.

## Discussion

Diffuse peritoneal carcinomatosis usually represents the advanced stage of transcoelomic dissemination from an intra-abdominal primary tumor, most commonly of Müllerian origin, such as HGSC of the ovary or fallopian tube, or from gastrointestinal adenocarcinomas. In this process, malignant epithelial cells exfoliate from the primary lesion, circulate within the peritoneal fluid, and implant on serosal surfaces, including the omentum, mesentery, diaphragm, and pelvic peritoneum. After adhering to mesothelial cells, they invade the submesothelial stroma and develop into metastatic deposits that secrete proangiogenic mediators, particularly vascular endothelial growth factor (VEGF), which increases vascular permeability, promotes protein-rich ascites, and contributes to diffuse peritoneal thickening and the characteristic appearance of omental caking. These features typically reflect metastatic spread from an identifiable primary tumor with a recognizable Müllerian immunophenotype. In such cases, carcinomatosis represents advanced metastatic disease from an identifiable primary tumor, which often demonstrates a Müllerian immunophenotype (e.g., PAX8 and WT1 positivity in serous ovarian carcinoma) [7].

There are, however, atypical presentations in which no ovarian or tubal mass is identified, as seen in PPSC, where carcinomatosis represents the primary disease process from the outset. Despite the absence of a detectable adnexal tumor, these neoplasms demonstrate Müllerian marker expression, including PAX8, CK7, WT1, and aberrant TP53, supporting their origin from Müllerian-type epithelium either within the peritoneum or through early exfoliation from the distal fallopian tube before formation of a clinically detectable mass [8]. Because dissemination occurs at a microscopically early stage, the original focus may remain occult, regress, or never evolve into a dominant tumor. As a result, patients may present with massive ascites, extensive carcinomatosis, and markedly elevated CA 125 levels while the ovaries appear normal, creating the clinical impression of a malignancy without a primary despite clear molecular evidence of Müllerian lineage [9].

Ascitic fluid in Müllerian carcinomatosis is typically an exudate containing abundant exfoliated malignant cells arranged in three-dimensional clusters, papillary fragments, or gland-like structures. These cells exhibit marked atypia, including hyperchromatic pleomorphic nuclei, prominent nucleoli, and frequent mitoses, and psammoma bodies may be present. Because mucin production is usually limited, immunocytochemistry plays a central role in determining tumor origin. The characteristic Müllerian profile includes positivity for CK7, PAX8, WT1, and abnormal p53 staining patterns, which facilitates distinction from gastrointestinal adenocarcinomas that commonly express CK20 and CDX2, and from malignant mesothelioma, which is defined by positivity for calretinin, D2 40, and CK5/6 [10].

The diagnosis and preoperative assessment of peritoneal carcinomatosis depend heavily on imaging. Common radiologic findings include ascites, peritoneal nodularity, mesenteric infiltration, and omental thickening. Ultrasound, although limited in sensitivity, is often the initial modality and is valuable for guiding procedures. Computed tomography is the primary imaging tool for staging, with sensitivity ranging from 79 to 86% and specificity from 82 to 89%, although accuracy decreases for lesions smaller than one centimeter or those in anatomically complex locations. Magnetic resonance imaging provides comparable or superior sensitivity, particularly for subcentimeter implants or lesions in the subphrenic regions. Accurate interpretation of imaging studies and use of tools such as the Peritoneal Carcinomatosis Index are essential for assessing resectability and selecting appropriate candidates for surgery or neoadjuvant therapy. Multidisciplinary evaluation remains critical for optimal decision making and coordinated management in this complex disease setting [11].

In the present case, the disease manifested with massive ascites and diffuse peritoneal carcinomatosis but without an adnexal mass, a presentation strongly suggestive of PPSC but challenging to differentiate clinically from other advanced malignancies. Despite the extensive peritoneal involvement, both ovaries and fallopian tubes were normal on gross and microscopic examination, which supports a primary peritoneal origin according to established diagnostic criteria. The diagnosis relied entirely on ascitic fluid cytology, which repeatedly demonstrated malignant epithelial cells with a Müllerian immunoprofile, including PAX8 positivity, CK7 expression, and an abnormal p53 pattern. Although imaging demonstrated diffuse peritoneal involvement, including omental thickening and mesenteric reticulation consistent with carcinomatosis, no discrete, safely accessible lesion was identified for targeted biopsy. Therefore, tissue biopsy was not performed, and the diagnosis was established based on repeated cytologic findings and consistent immunophenotypic features. Magnetic resonance imaging (MRI) was not pursued, as CT provided sufficient evaluation of disease extent and is the standard first-line imaging modality in this clinical setting. This allowed accurate tumor classification despite the absence of a detectable primary mass and guided appropriate oncologic management.

The degree of pathologic response after neoadjuvant chemotherapy is an important prognostic factor in high-grade serous Müllerian carcinoma, as greater treatment response has been associated with improved survival outcomes [12,13]. True pathologic complete response is uncommon and typically observed in only 5 to 10% of patients [12]. Response is often assessed using the Chemotherapy Response Score, which categorizes treatment effect into CRS 1 with minimal response, CRS 2 with partial response, and CRS 3 with complete or near-complete response, characterized by the absence of viable tumor or only minimal scattered microscopic foci. CRS 3 is reported in 20 to 28% of cases and is strongly associated with significantly improved progression-free and overall survival [13]. In this patient, the normalization of CA-125, complete radiologic resolution of disease, and absence of viable carcinoma on final pathology are consistent with a pathologic complete response (CRS 3). This represents an exceptional and clinically meaningful outcome given the diffuse carcinomatosis at presentation.

This exceptional response also raises consideration of factors that may influence variability in treatment efficacy. Tumor biology plays a central role, as chemosensitivity in high-grade serous Müllerian carcinomas is closely associated with genomic instability and defects in homologous recombination pathways, including BRCA1/2 alterations [14]. Clinical factors such as baseline disease burden and overall tumor distribution may also affect treatment response and drug delivery. In primary peritoneal carcinoma, the absence of a dominant ovarian mass may reflect a distinct pattern of dissemination, although its impact on therapeutic response remains incompletely understood. These considerations highlight the heterogeneity of treatment outcomes and underscore the importance of integrating both biological and clinical factors when interpreting response to platinum-based chemotherapy [14].

In patients with extensive peritoneal dissemination or a low likelihood of achieving optimal primary cytoreduction, neoadjuvant chemotherapy followed by interval debulking surgery is a well-supported strategy [15]. Large randomized trials, including EORTC 55971 and CHORUS, have shown that this approach provides overall and progression-free survival comparable to primary cytoreductive surgery while reducing perioperative morbidity, particularly in high-risk or unresectable cases [16]. Current ASCO and NCCN guidelines therefore recommend neoadjuvant therapy for women with bulky disease, poor performance status, or low probability of complete primary cytoreduction after evaluation by a gynecologic oncologist [17]. Although the TRUST trial later suggested that primary surgery may offer improved progression-free survival in carefully selected non-frail patients treated at expert centers, neoadjuvant chemotherapy remains the preferred option when disease burden is high or resectability is limited [18]. In this case, the patient’s diffuse carcinomatosis, ascites, and absence of a dominant mass supported neoadjuvant platinum-based therapy, which enabled successful interval debulking with no residual disease.

The addition of bevacizumab to neoadjuvant chemotherapy in advanced ovarian and primary peritoneal carcinoma continues to be investigated. Phase II data from the ANTHALYA trial suggest that incorporating bevacizumab may increase complete resection rates without adding significant perioperative morbidity, although the GEICO 1205 trial showed no clear improvement while confirming safety and feasibility [19]. Due to potential interference with wound healing, guidelines emphasize holding bevacizumab for four to six weeks before interval surgery. In frontline treatment, evidence from ICON7 and GOG 218 demonstrates that bevacizumab provides the greatest benefit in high-risk patients, including those with stage IV disease or incomplete cytoreduction, especially when continued as maintenance therapy [18,19]. 

In this patient, the incorporation of bevacizumab during neoadjuvant chemotherapy was clinically justified given the extensive disease burden, and was administered with appropriate perioperative timing. This allowed safe interval debulking without complications and contributed to her excellent overall response. The subsequent complete radiologic resolution further supports the real-world effectiveness of combining platinum taxane chemotherapy with antiangiogenic therapy in selected patients with primary peritoneal carcinoma.

This case offers several important contributions to the existing literature on Müllerian peritoneal carcinomatosis. First, it highlights the diagnostic challenge posed by diffuse peritoneal involvement in the absence of an adnexal mass, emphasizing that cytologic evaluation and Müllerian immunophenotyping can provide definitive diagnostic clarity even when imaging fails to identify a primary tumor. Second, it demonstrates the clinical value of ascitic fluid analysis as the sole diagnostic tissue source, reinforcing its reliability in cases where biopsy of a solid lesion is not feasible. Third, the patient’s exceptional response to neoadjuvant therapy, culminating in complete biochemical, radiologic, and pathologic remission, represents an outcome achieved by only a minority of patients with high-grade serous carcinoma and underscores the prognostic importance of achieving a CRS 3 or pathologic complete response. Finally, the safe and effective incorporation of bevacizumab in the neoadjuvant setting illustrates the feasibility of antiangiogenic therapy in patients with extensive peritoneal disease when perioperative timing is carefully managed. Collectively, this case underscores the importance of individualized treatment planning, highlights the potential for remarkable therapeutic response even in widely disseminated disease, and adds meaningful real-world evidence to the spectrum of clinical presentations and outcomes in primary peritoneal serous carcinoma.

A limitation of this report is the absence of histologic confirmation from a solid tumor, as the diagnosis relied solely on ascitic cytology. Although imaging demonstrated diffuse peritoneal involvement, no discrete, safely accessible lesion was identified for targeted biopsy, which limited the feasibility of obtaining tissue for histopathologic confirmation. This may restrict full architectural characterization of the malignancy. Additionally, as a single case, the exceptional treatment response cannot be generalized. Nonetheless, the consistent Müllerian immunoprofile and surgical findings strongly support the diagnosis and clinical relevance.

## Conclusions

This case highlights an uncommon presentation of primary peritoneal high-grade serous carcinoma, characterized by diffuse peritoneal carcinomatosis without an ovarian mass and diagnosed exclusively through ascitic cytology supported by Müllerian immunophenotyping. It underscores the importance of recognizing that, in the absence of a detectable primary tumor, cytologic analysis of ascitic fluid can provide definitive diagnostic clarity and prevent diagnostic delay. The case also illustrates the need to distinguish this entity from advanced ovarian carcinoma, gastrointestinal malignancies, and mesothelioma, emphasizing the diagnostic value of an appropriate immunohistochemical panel.

Furthermore, this patient demonstrated an exceptional therapeutic response, achieving a complete biochemical, radiologic, and pathologic response following neoadjuvant platinum-taxane chemotherapy with bevacizumab. Such outcomes are rare in high-grade Müllerian carcinomas and reinforce the prognostic significance of tumor chemosensitivity and individualized treatment selection. This case also highlights the potential role of underlying tumor biology and other clinical factors in influencing variability in treatment response. By documenting a rare presentation paired with an unusually robust therapeutic response, this case expands the clinical understanding of PPSC, highlights the potential effectiveness of neoadjuvant therapy in appropriately selected patients, and contributes meaningful real-world evidence to the management of advanced Müllerian peritoneal malignancies.
